# Pediatric Priapism: A Rare First Manifestation of Leukemia

**Published:** 2013-10-18

**Authors:** Shankar Prasad Hazra, Vinod Priyadarshi, Debojit Gogoi, Pramod Kumar Sharma, Dilip Kumar Pal, Sudip C Chakraborty

**Affiliations:** Department of Urology, IPGMER and SSKM Hospital, Kolkata-20, India.

**Keywords:** Priapism, Leukemia, Child

## Abstract

Priapism is a rare disease. It is an emergency condition with a poor prognosis, and the risk of impotence is 50% despite appropriate management. Though about 20% cases of all priapism are related to hematological disorders, the incidence of priapism in adult leukemic patients is only about 1-5 percent. The incidence in pediatric leukemia patient is even rarer. Here we present a case of priapism in a 14-year-old apparently healthy boy who found to have chronic myeloid leukemia on subsequent investigations.

## INTRODUCTION

Priapism is a full or partial erection that continues more than 4 hours beyond sexual stimulation and orgasm or is unrelated to sexual stimulation.[1] It is rare in children and occurs mostly in patients with hemoglobinopathy.[2] A sole presentation of priapism in an apparently healthy child leading to diagnosis of leukemia, is exceptional. One such case is reported.

## CASE REPORT

A young healthy 14-year-old boy was referred from a local hospital for persistent painful erection of the penis for approximately 24 hours. There was no history of sexual stimulation, trauma, previous similar episodes, use of medications or any chronic illness. On examination he had pallor. The spleen was palpable 6 cm below the left costal margin. Penis was erect, firm, and tender with prominent superficial veins. Urinalysis was normal. Hemoglobin was 9.9 g/dL, hematocrit 28%, white blood count 2,26,900/ and platelets 3,10,000/ uL. A peripheral blood smear demonstrated immature leukocytes in various stages of differentiation (blast cell 10%, myelocyte 10% and metamyelocyte 25%). Bone marrow analysis was suggestive of chronic myeloid leukemia (CML) (Fig. 1). Color doppler ultrasonography suggested a low flow priapism while cavernosal blood gas analysis revealed acidosis with hypercarbia (pH=7.2, Pco2 = 93, Po2= 27). This suggested an ischemic type of priapism. Initial management done by cavernosal aspiration, irrigation and phenylepinephrine injection that achieved partial detemusence with reduction of pain. At the same time, patient was supported with intravenous fluid hydration and moist oxygen inhalation. After obtaining the bone marrow analysis, hydroxyurea (50 mg/kg/day) along with allopurinol (300 mg/day) were also added. After 5 days of therapy priapism reduced to normal flaccid state of penis. Patient is presently receiving chemotherapy. There was no further episode of priapism over the last 2 months.

**Figure:**
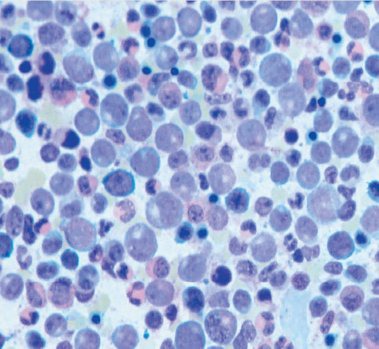
Figure 1:Bone marrow smear showing hyper-cellularity with myeloid hyperplasia.

## DISCUSSION

The term priapism has its origin in reference to the Greek god Priapus, who was worshipped as a god of fertility and had had a disproportionate permanent erection.[2] Priapism is caused by imbalance of penile blood inflow and outflow and it may be either low-flow (ischemic) or high-flow (non-ischemic) type. Low-flow or ischemic priapism results from venous occlusion and manifests as a painful, rigid erection. Intracavernosal blood sampling in such cases show acidosis (pH<7.25), hypercarbia (Pco2 >90) and a decrease in oxygen tension (Po2<30).[1] This type is more common and is an emergency because irreversible cellular damage and fibrosis can occur if treatment is not administered within 24 to 48 hours.[3] It results in long term squeal of erectile dysfunction or predisposition to frequent, prolonged episodes of priapism.[4] The cause of low-flow priapism are idiopathic, hematologic disorders, tumor infiltrate, or drug induced.[1,5] High-flow or arterial priapism differs in that it results from increased arterial inflow into the cavernosal sinusoids, which overwhelms venous outflow and clinical presentation is usually painless and less rigid.[1] In contrast to low-flow priapism, intracavernosal blood sampling from patients with high-flow priapism reveal bright red oxygenated blood (pH =7.4, Pco2<40, Po2>90).[5] Irreversible cellular damage and fibrosis are rare in this variety.[3] This type of priapism is usually occurs following trauma that results in injury to the cavernosal or helician artery which establishes a fistula between the cavernosal artery and the corpus cavernosum and an unregulated inflow occurs.[6]

Though a rare event in children, the most common cause for priapism in this population is sickle cell anemia (67%) while leukemia accounts for 15% of such cases.[2,5] Chronic myeloid leukemia is responsible for 50% of cases of leukemic priapism although it is also observed in acute myeloblastic and lymphoblastic leukemias.[2] The incidence of priapism in adult patients with leukemia is about 1–5%. In pediatric patients it is uncommon.[2,7] Also a presentation of priapism as a first manifestation of leukemia is highly exceptional.[2,8] The same was observed in the index case.

Leukemic priapism is mostly low flow ischemic type and caused by hyper-leukocytosis. This may result from aggregation of leukemic cells in the corpora cavernosa and the dorsal veins of penis or from venous congestion of the corpora cavernosa due to mechanical pressure on the abdominal veins by the splenomegaly. Alternately infiltration of the sacral nerves with leukemic cells or infiltration of the central nerve system may be the cause.[2,9]

Differentiation from non-ischemic variety must be done and apart from history and clinical examination, cavernosal blood gas analysis and color duplex ultrasonography are currently the most reliable diagnostic methods of distinguishing ischemic from nonischemic priapism.[1] Initial penile intervention may utilize therapeutic aspiration with or without irrigation. If priapism persists even after aspiration/irrigation, intracavernous injection of sympathomimetic drugs should be performed. Surgical shunting should be the last resort if detumescence is not achieved in 24-48 hours. The purpose of shunt is to establish a new venous outflow and restore normal arterial flow to the corpora cavernosa.[1] In the present case, combined systemic and surgical intervention successfully achieved detumescence without shunt creation. Priapism is an uncommon presentation in CML that all physicians should be aware of.

## Footnotes

**Source of Support:** Nil

**Conflict of Interest:** None declared

